# Quantitative Structure—Permittivity Relationship Study of a Series of Polymers

**DOI:** 10.1021/acsmaterialsau.3c00079

**Published:** 2024-01-09

**Authors:** Yevhenii Zhuravskyi, Kweeni Iduoku, Meade E. Erickson, Anas Karuth, Durbek Usmanov, Gerardo Casanola-Martin, Maqsud N. Sayfiyev, Dilshod A. Ziyaev, Zulayho Smanova, Alicja Mikolajczyk, Bakhtiyor Rasulev

**Affiliations:** † Department of Technology of Organic Products, Lviv Polytechnic National University, Lviv 79013, Ukraine; ‡ Department of Coatings and Polymeric Materials, North Dakota State University, Fargo, North Dakota 58102, United States; § Institute of the Chemistry of Plant Substances AS RUz, Tashkent 100170, Uzbekistan; ∥ Department of Chemistry, National University of Uzbekistan, Tashkent 100174, Uzbekistan; ⊥ Laboratory of Environmental Chemometrics, Institute for Environmental and Human Health Protection, Faculty of Chemistry, University of Gdansk, Gdansk 80-308, Poland

**Keywords:** dielectric permittivity, polarization, polymers, descriptors, models, machine learning

## Abstract

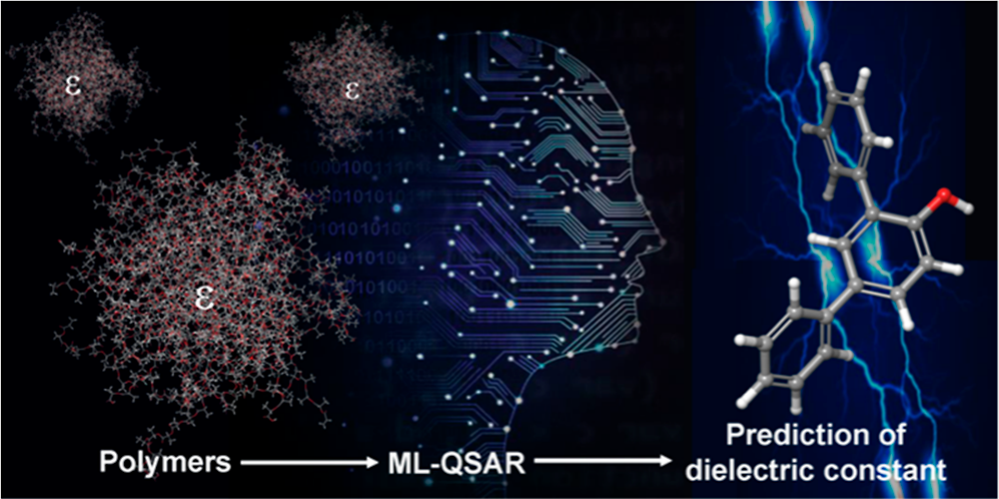

Dielectric constant is an important property which is widely utilized in many scientific fields and characterizes the degree of polarization of substances under the external electric field. In this work, a structure–property relationship of the dielectric constants (ε) for a diverse set of polymers was investigated. A transparent mechanistic model was developed with the application of a machine learning approach that combines genetic algorithm and multiple linear regression analysis, to obtain a mechanistically explainable and transparent model. Based on the evaluation conducted using various validation criteria, four- and eight-variable models were proposed. The best model showed a high predictive performance for training and test sets, with *R*^2^ values of 0.905 and 0.812, respectively. Obtained statistical performance results and selected descriptors in the best models were analyzed and discussed. With the validation procedures applied, the models were proven to have a good predictive ability and robustness for further applications in polymer permittivity prediction.

## Introduction

Polymeric properties related to electrical conductivity are useful in many applications, such as cable insulation,^1^ capsules for electrical components, interlayer dielectrics, charge-storage capacitors,^2,3^ and printed circuit boards.^4^ Dielectric permittivity is an important value that is widely used and characterizes the degree of polarization of substances under the action of an external electric field. A larger dielectric constant means a larger polarization of the medium between the two charges. Therefore, the dielectric constant is the ability of a substance to separate the charge or orient its molecular dipoles in an external electric field. The dielectric constant is an important basic molecular property that can also be a useful predictor of other electrical properties of polymers.^4–6^ However, the exact experimental values of the dielectric constant for polymers are often unavailable. The prediction of dielectric constants computationally and by using theoretical approaches, such as machine learning predictive modeling, is important in the molecular design of new polymeric materials with the desired properties. The rapid and accurate implementation of predictions for a wide variety of chemical structures can significantly improve the performance and speed of phenomena investigation. However, the theoretical calculation of the property, such as dielectric constant of the polymer is not an easy problem, because this property is a nonlinear property and, therefore, a function of several factors, including polymer structure and composition, temperature, materials morphology, additives and plasticizers, impurities, and moisture in the volume of the polymer. A quantitative structure–activity/property relationship (QSAR/QSPR) is a subsection of machine learning (ML) modeling and chemical informatics for revealing relationships between chemical structures of molecules and their activity. QSAR modeling is a suitable approach for estimating the properties of polymers based on numerical features/descriptors derived from the molecular structure to fit the experimental data.^7–9^ The main idea of the QSAR approach is that the change in the desired property of a compound can be correlated with the structure-based properties that numerically expressed and called “molecular descriptors”.^8–11^ In cheminformatics, molecular descriptors are numbers that formally represent a molecule, obtained by a well-defined algorithm and applied to a well-defined experimental procedure. In other words, a molecular descriptor is the result of a mathematical expression that converts the chemical structure to a numerical value.^12^ Each molecular descriptor describes a molecular structure by encoding a part of the structure or a whole molecular structure. Molecular descriptors play a fundamental role in the development of QSPR models. One of the main features of the QSPR approach is that it requires only knowledge of the chemical structure and is independent of any experimental properties. Once a correlation is found, it can be applied to predict the properties of new compounds/materials that have not been synthesized previously or not found. Therefore, the QSPR approach can accelerate the development of new molecules and materials with the required properties. Using the QSPR approach, many different properties of polymers can be determined with a sufficient accuracy, in particular, this approach is already used to determine, such properties as a refractive index,^4,13–21^ glass transition temperature,^14,22–33^ cohesive energy,^34^ thermal decomposition temperature,^35^ solubility parameter,^36^ as well as fouling release properties.^37^ Several QSPR models for the dielectric constants of small organic molecules have also been reported in the literature.^6,38–41^ But the number of attempts to predict the dielectric constants of polymers was rather small.^4,42^ Liu et al.^42^ introduced a model with a correlation coefficient of (*R*^2^) 0.908 and a standard error (s) of 0.001 for 22 polyalkenes using three descriptors, but the values of ε in this case cover only the range from 2.154 to 2.165. Bicerano^4^ developed a QSPR model with (*R*^2^) 0.958 and (s) 0.087 to correlate ε with 32 topological and constitutional descriptors for 61 polymers. This model is good but contains too many descriptors. High correlation and randomness of correlations may be partly due to increased number of descriptors in the model and use a whole dataset as a trainig set. Moreover, the two models were not validated externally using a test set. In fact, validation is a crucial aspect of any QSPR/QSAR modeling.^43^

The purpose of this study was to develop a reliable predictive QSPR model that could effectively be used to predict dielectric constant values with mechanistically explainable descriptors for further design applications. The model is developed using a set of 71 polymers with a large structural diversity, with further model validation applying specific validation approaches and an external set.

## Materials and Methods

### Data Set

The experimental data (polymers 1–56) were taken from the source that published by Bicerano,^4^ the remaining data (polymers 57–71) from the source published by Ku and Liepins,^5^ at room temperature (298 K). In total, the data set for this study consists of 71 polymers with diverse structures (see Table 1). The data set contains polymers of the following types: polyvinyls, polyethylenes, polyoxides, polystyrenes, polyethers, polysulfones, polyacrylnitrile, polyamides, polyacrylates, poly siloxanes, polyxylylenes, and polycarbonates.

**Table 1:** Set of Experimental and Predicted Dielectric Constant Data for the Polymers Involved in the Experiment

				Equation 1.		Equation 2.	
no	name	data set status	Exp.	Pred.	residual	pred.	residual
1	poly(1,4-butadiene)	train.	2.51	2.4104	–0.0996	2.6006	0.0906
2	poly[oxy(2,6-dimethyl-1,4-phenylene)]	train.	2.6	2.9651	0.3651	2.7210	0.1210
3	bisphenol-A polycarbonate	train.	2.9	3.0325	0.1325	2.8725	–0.0275
4	poly(ether ketone)	train.	3.2	3.0998	–0.1002	3.0820	–0.1180
5	poly(ethylene terephthalate)	train.	3.25	3.1291	–0.1209	3.0958	–0.1542
6	poly(chloro-*p*-xylylene)	train.	2.95	2.8054	–0.1446	2.7932	–0.1568
7	polyacrylonitrile	train.	4	3.6164	–0.3836	3.9567	–0.0433
8	polystyrene	train.	2.55	2.4631	–0.0869	2.3794	–0.1706
9	polypropylene	train.	2.2	2.3304	0.1304	2.3763	0.1763
10	poly(*p*-xylylene)	train.	2.65	2.4154	–0.2346	2.3772	–0.2728
11	polyisobutylene	train.	2.23	2.1490	–0.0810	2.2123	–0.0177
12	poly(*p*-chloro styrene)	train.	2.65	2.8016	0.1516	2.7449	0.0949
13	poly(*N*-vinyl carbazole)	train.	2.9	2.9390	0.0390	2.7868	–0.1132
14	poly(vinyl cyclohexane)	train.	2.25	2.3931	0.1431	2.1312	–0.1188
15	polyisoprene	test	2.37	2.2119	–0.1581	2.4058	0.0358
16	poly(p-hydroxybenzoate)	train.	3.28	3.1280	–0.1520	3.1413	–0.1387
17	poly(vinyl butyral)	train.	2.69	2.9227	0.2327	3.0580	0.3680
18	poly(cyclohexyl methacrylate)	train.	2.58	2.9625	0.3825	2.7652	0.1852
19	poly(vinyl acetate)	train.	3.25	2.9128	–0.3372	3.1751	–0.0749
20	poly(e-caprolactam)	train.	3.5	3.5411	0.0411	3.4218	–0.0782
21	poly(3,4-dichlorostyrene)	test	2.94	2.7643	–0.1757	2.9000	–0.0400
22	poly(hexamethylene adipamide)	train.	3.5	3.5852	0.0852	3.5226	0.0226
23	poly(hexamethylene sebacamide)	test	3.2	3.5443	0.3443	3.3880	0.1880
24	poly(isobutyl methacrylate)	train.	2.7	2.8675	0.1675	2.7456	0.0456
25	poly(vinyl chloride)	train.	2.95	3.1896	0.2396	2.9759	0.0259
26	poly(*m*-chloro styrene)	train.	2.8	2.6153	–0.1847	2.8629	0.0629
27	polychlorotrifluoroethylene	test	2.6	2.1061	–0.4939	2.2365	–0.3635
28	poly(ethyl methacrylate)	train.	3	2.8124	–0.1876	2.8927	–0.1073
29	poly(*n*-butyl methacrylate)	test	2.82	2.9877	0.1677	2.9430	0.1230
30	poly(methyl methacrylate)	train.	3.1	2.8846	–0.2154	2.8868	–0.2132
31	poly[2,2′-(m-phenylene)-5,5′-bibenzimidazole]	train.	3.3	3.3864	0.0864	3.4484	0.1484
32	polyethylene	test	2.3	2.4908	0.1908	2.3596	0.0596
33	poly(*a*-vinyl naphthalene)	test	2.6	2.4277	–0.1723	2.4579	–0.1421
34	poly(tetramethylene terephthalate)	train.	3.1	3.2794	0.1794	3.1749	0.0749
35	poly[thio(*p*-phenylene)]	train.	3.1	3.4506	0.3506	3.2558	0.1558
36	poly(4-methyl-1-pentene)	train.	2.13	2.1958	0.0658	2.2527	0.1227
37	poly(1-butene)	train.	2.27	2.3378	0.0678	2.4632	0.1932
38	poly(a,a,a′,a′-tetrafluoro-p-xylylene)	train.	2.35	2.4386	0.0886	2.4448	0.0948
39	poly(*o*-methylstyrene)	train.	2.49	2.4046	–0.0854	2.4385	–0.0515
40	poly(*b*-vinyl naphthalene)	train.	2.51	2.4622	–0.0478	2.5002	–0.0098
41	poly(*a*-methylstyrene)	test	2.57	2.4189	–0.1511	2.3614	–0.2086
42	poly[oxy(2,6-diphenyl-1,4-phenylene)]	train.	2.8	2.8875	0.0875	2.9843	0.1843
43	poly(vinylidene chloride)	train.	2.85	2.9919	0.1419	2.7289	–0.1211
44	poly(*p*-methoxy-*o*-chloro styrene)	train.	3.08	3.0777	–0.0023	3.1591	0.0791
45	poly(ethyl a-chloroacrylate)	test	3.1	3.1639	0.0639	3.4555	0.3555
46	poly(methyl a-chloroacrylate)	train.	3.4	3.2357	–0.1643	3.4685	0.0685
47	poly(oxy-2,2-dichloromethyltrimethylene)	train.	3	3.1478	0.1478	3.0166	0.0166
48	Ultem 1000	test	3.15	3.4652	0.3152	3.3747	0.2247
49	polyoxymethylene	train.	3.1	2.9951	–0.1049	3.0158	–0.0842
50	poly(1,4-cyclohexylidene dimethylene terephthalate)	train.	3	3.1045	0.1045	3.0271	0.0271
51	poly[*N*,*N′*-(*p*,*p*′-oxidiphenylene)pyromellitimide]	train.	3.5	3.5482	0.0482	3.5032	0.0032
52	poly[4,4′-diphenoxy di(4-phenylene)sulfone]	train.	3.44	3.3943	–0.0457	3.4010	–0.0390
53	poly[4,4′-isopropylidene diphenoxy di(4-phenylene)sulfone]	test	3.18	3.3082	0.1282	3.4309	0.2509
54	poly[4,4′-sulfone diphenoxy di(4-phenylene)sulfone]	train.	3.8	3.5963	–0.2037	3.6851	–0.1149
55	poly[1,1-cyclohexane bis(4-phenyl)carbonate]	test	2.6	3.0918	0.4918	3.0117	0.4117
56	poly[1,1-ethane bis(4-phenyl)carbonate]	train.	2.9	3.0499	0.1499	2.9578	0.0578
57	poly(cellulose propionate)	train.	3.2	3.1174	–0.0826	3.4035	0.2035
58	poly(amide-imide)	train.	3.32	3.4811	0.1611	3.3832	0.0632
59	poly(diallyl phthalate)	train.	3.57	3.2808	–0.2892	3.3366	–0.2334
60	poly(diallyl phenyl phosphonate)	train.	3.84	3.6409	–0.1991	3.7696	–0.0704
61	poly(2,5-dichlorostyrene)	train.	2.61	2.7786	0.1686	2.8859	0.2759
62	polyfumaronitrile	excl.	8.5				
63	poly(methyl cellulose)	excl.	6.8				
64	Nylon-11	train.	3.3	3.3744	0.0744	3.3483	0.0483
65	Nylon-12	train.	3.6	3.3392	–0.2608	3.3367	–0.2633
66	poly(vinyl fluoride)	excl.	8.5				
67	poly(2-vinylpyridine)	excl.	4.64				
68	poly(vinyl toluene)	train.	2.59	2.4524	–0.1376	2.4418	–0.1482
69	poly(vinylidene fluoride)	excl.	8.4				
70	poly(dichloro-*p*-xylylene)	test	2.82	2.9745	0.1545	2.8109	–0.0091
71	poly(methyl-*p*-xylylene)	train.	2.48	2.3989	–0.0811	2.4105	–0.0695

### Computational Details

**Figure 1:**
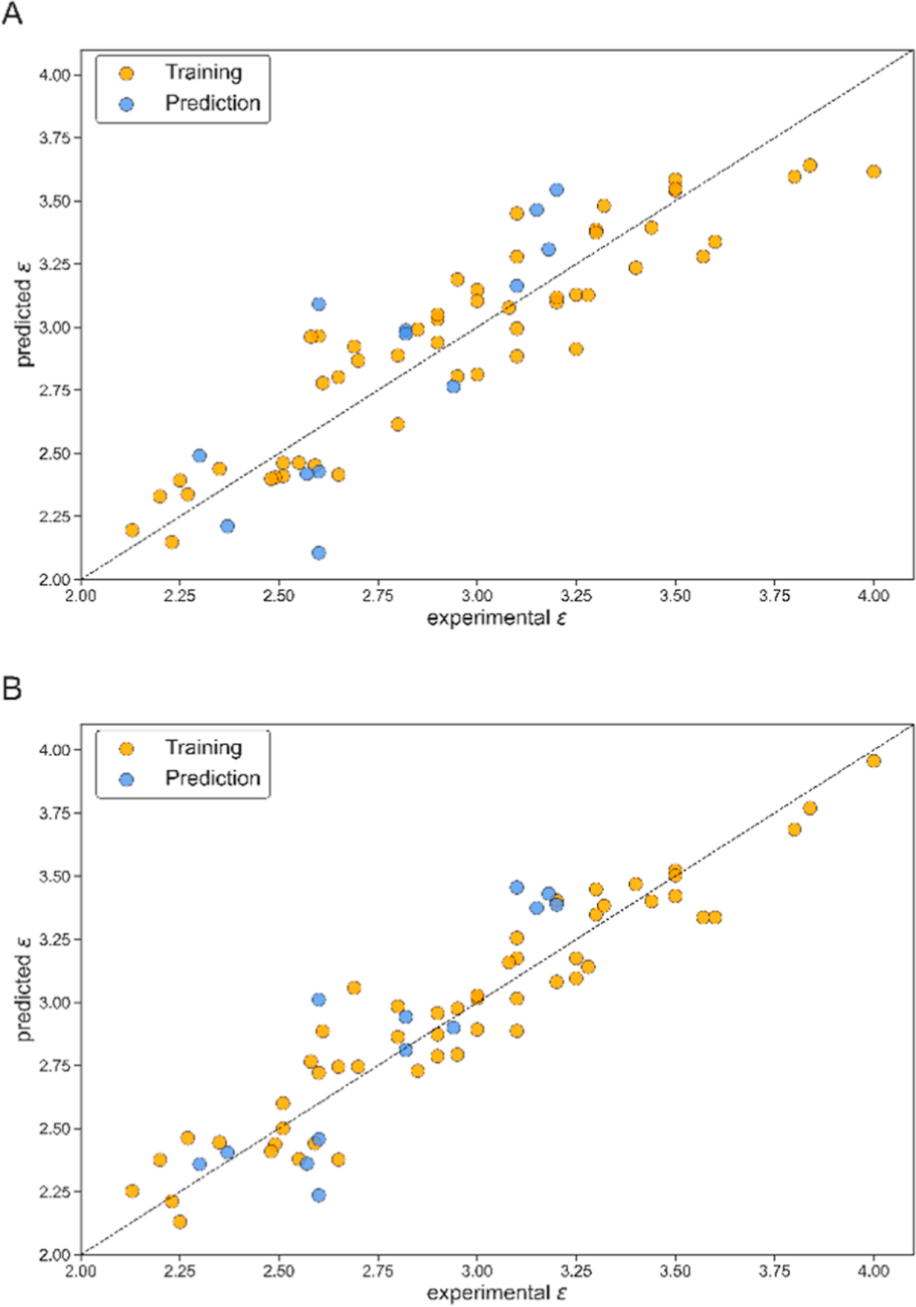
Plots of experimental and predicted values of the dielectric constants for the entire data set. Yellow dots are the training set, and blue dots are the test set (A—for eq 1.; B—for eq 2).

In this work, the structures of all polymers were computationally optimized and used for generating structural properties/features/descriptors calculation. Because polymers are macromolecules with a large size and wide chain length distribution, the calculation of structural descriptors based on original structural formulas was not possible using current descriptor-generating software.^23,30^ Moreover, due to the high molecular weight of the polymers, the effect of the terminal groups on the overall structure of polymer is quite small, which allows us to neglect the contribution of the terminal structure contribution. In this regard, the structures of repeating monomer units of investigated polymers were used to calculate the structural features/descriptors (as shown in Figure 1).^13,15,22–24,30^ We assumed that the main contributing factor to the polymer property is the structure of monomer units and, therefore, the molecular descriptors are calculated based on the structure of repeating monomer units.^44,45^

The molecular structures of each polymer were drawn in ChemSketch software.^46^ The optimization of monomeric units, i.e., geometry optimization and finding the minimal energy conformation, is an important step and provides a real conformation of the investigated structure for further QSAR modeling. Molecular modeling is often used for optimization and property assessment of various chemical systems.^47–50^ In this work, the geometry optimization was carried out using HyperChem software, applying molecular mechanics force-field MM+.^51^ The criterion for the energy optimization limit was chosen as the achieved gradient of 0.01 kcal/mol. The molecular descriptors for each polymer were calculated based on minimal energy conformation using DRAGON software.^52^ Dragon 6.0 allows one to generate about 5000 descriptors per structure.^52^ The generated descriptors include the following categories: constitutional indices, 2D and 3D matrix-based descriptors, 2D autocorrelations, topological descriptors, indicator descriptors, connectivity indexes, information indices, atom-centered fragments, charge-based descriptors, 0D, 2D, and 3D descriptors, molecular properties, and so on.^12^ Descriptors with high correlations, single variables, and noninformative information were discarded based on the constant value, near constant (*R* > 0.95), and pair correlation criteria (*R* > 0.7).

A total of 523 descriptors of different types were selected from about 5000 descriptors after the initial filter criteria applied. Each descriptor represents a molecular graph invariant, describes the particular property, and overall adds to chemical diversity of the monomeric unit.

The model development was performed by QSARINS software^54,55^ with the following setup to find the best model. For the genetic algorithm (GA)-based variable selection step, the number of generations was set to 2000 and a mutation rate of 35% was used. For the best models’ selection, the population size of the final models’ list was set to 20. For validation purposes, multiple methods were applied, including leave-one-out (LOO) cross validation, *y*-scrambling, as well as internal and external validation protocols. After validation techniques were applied, the best model was chosen based on multiple criteria: (1) high statistical performance of *R*^2^ and *Q*^2^ variables (including *R*^2^ – *Q*^2^ < 0.3);^43^ (2) a low number of variables in the model; (3) low cross-correlation between descriptors in the selected model; and (4) best performance of *R*^2^ for the external validation set (test set) to avoid model overfitting.^43^

## Results and Discussion

In this work, a data set of 71 polymers was used to develop a quantitative structure–permittivity relationship model. For the model validation, the set was split into training and test sets consisting of 57 (80%) and 14 (20%) polymers, respectively. The splitting was performed with care to ensure that at least one compound of each structural class in the training set was represented in the test set. After genetic algorithm combined with multiple linear regression analysis (GA-MLRA) computation iterations, the best models were found. After a first round of GA-MLRA it was found that five compounds are outliers, with a high prediction value error. The outliers are 62, 63, 66, 67, and 69. After elimination of outliers, the GA-MLRA iteration was repeated. The set with a total of 66 components was split into training and test sets containing 53 (80%) and 13 (20%) polymers, respectively. In the process of finding the best model, several options were selected that best correlate with the dielectric constants of the selected polymers. Two models with four and eight variables are proposed, the statistical characteristics of which are given in Table 2.

**Table 2:** Statistical Characteristics of the Four- and Eight-Variable Models

model	no. of descriptors	*R*_train_^2^	*R*_adj_^2^	*s*	*F*	*Q*^2^	*R*_test_^2^
1	4	0.842	0.829	0.187	64.124	0.813	0.715
2	8	0.905	0.888	0.151	52.542	0.865	0.812

The following equations represent the proposed models with four (1) and eight (2) variables
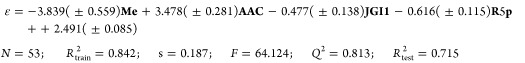
1
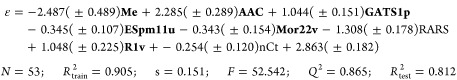
2

The four-variable model shows a good performance, with *R*_train_^2^ = 0.842 and *R*_test_^2^ = 0.715. A graphical representation of the model for the training and test sets is given in Figure 1A. Compared to the 4-variable model, the eight-variable model shows better *R*_train_^2^ and *Q*^2^ performance values for the training set, smaller standard deviation s, and better predictive performance due to higher *R*_test_^2^ for the test set, 0.812. In comparison to the four-variable model, the 8-variable model has a larger number of variables, which can lead to some level of overfitting, but still very robust. A graphical representation of the model for the training and test sets is presented in Figure 1B.

Both equations: (1) and (2) show satisfactory statistical results that confirm the robustness of these models. However, considering the combined productivity for both training and test sets, the second model provides a better performance.

Descriptor selection was performed by applying a variable selection GA algorithm, followed by the MLRA approach together with a cross-validation LOO procedure. Based on the size of the data set and the correlation coefficients of the training and test sets (*R*_train_^2^ and *R*_test_^2^), the significance criterion F and the standard errors, the number of descriptors in the final QSPR model was determined.

A very important step in the model's robustness is to check the applicability domain (AD). Predictions of compounds can be considered reliable only if the dataset’s chemical space of applicability is within the predictive chemical space of the developed model, before the model can be applied for further predictions. The AD check was performed by application of leverage approach, i.e., William’s plot evaluation for the final models. All data points were within the three standardized residues (±3σ) and within the HAT index, where h* is the critical value of leverage h. If the errors of estimation would exceed the values of the standardized residues, then the predicted values could go out of the AD and give inaccurate predictions as they go beyond reasonable extrapolation. If the value of *h* of the resulted data is higher than h*, then they are considered as structurally significant contributors to the model.^53^

**Figure 2:**
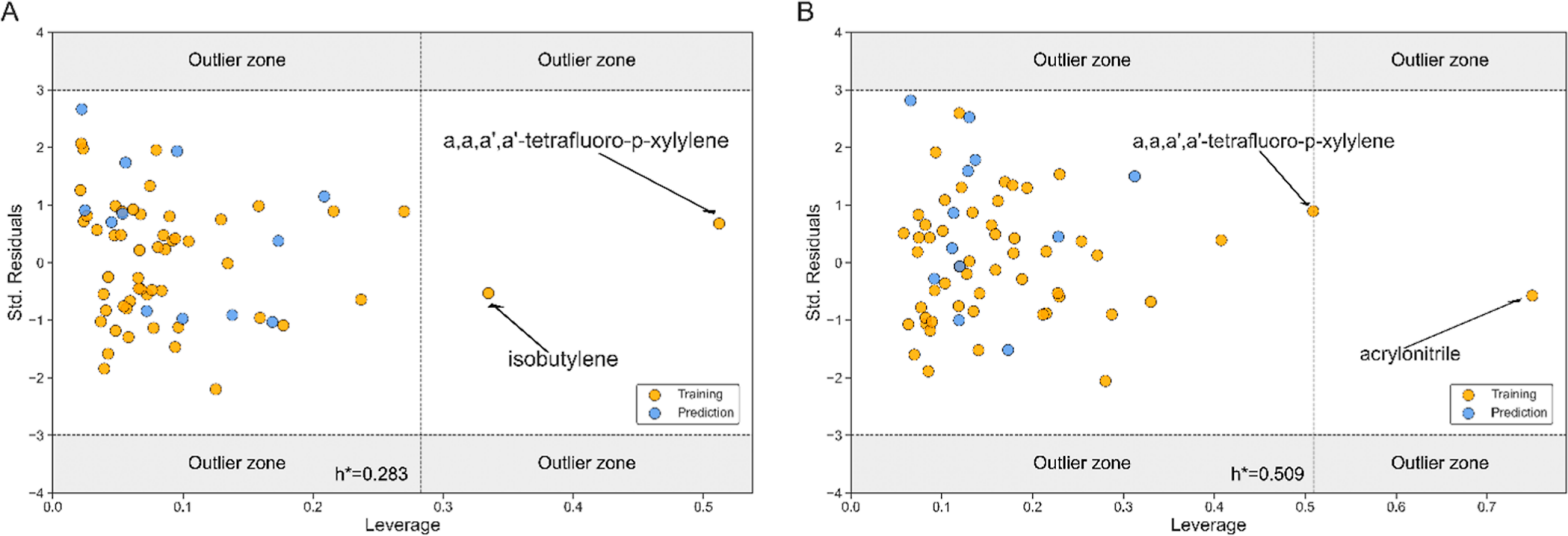
Williams plots for eq 1. (A) and eq 2. (B): yellow balls—training set; blue balls —test set.

As can be seen in the Williams plots (Figure 2) for both equations, in the first model (A) there are only two polymers, and in the second (B) only one polymer has values *h* higher than h*. However, these polymers have low residual values, which means that the model is stable enough to make reliable predictions for all polymers structurally similar to the ones in the data set.

The obtained models contain the following descriptors: Me—mean atomic Sanderson electronegativity (scaled on carbon atom); AAC—mean information index on atomic composition; R5p+—R maximal autocorrelation of lag 5/weighted by polarizability; JGI1—mean topological charge index of order 1; GATS 1p—Geary autocorrelation of lag 1 weighted by polarizability; Mor22v—signal 22/weighted by van der Waals volume; RARS—R matrix average row sum; ESpm11u—Spectral moment 11 from edge adj. matrix; R1v+—R maximal autocorrelation of lag 1/weighted by van der Waals volume; and *nCt*—number of total tertiary *C*(sp^3^).

More information about these descriptors can be found in the Dragon software user’s guide^12,52^ and the references therein.

**Figure 3:**
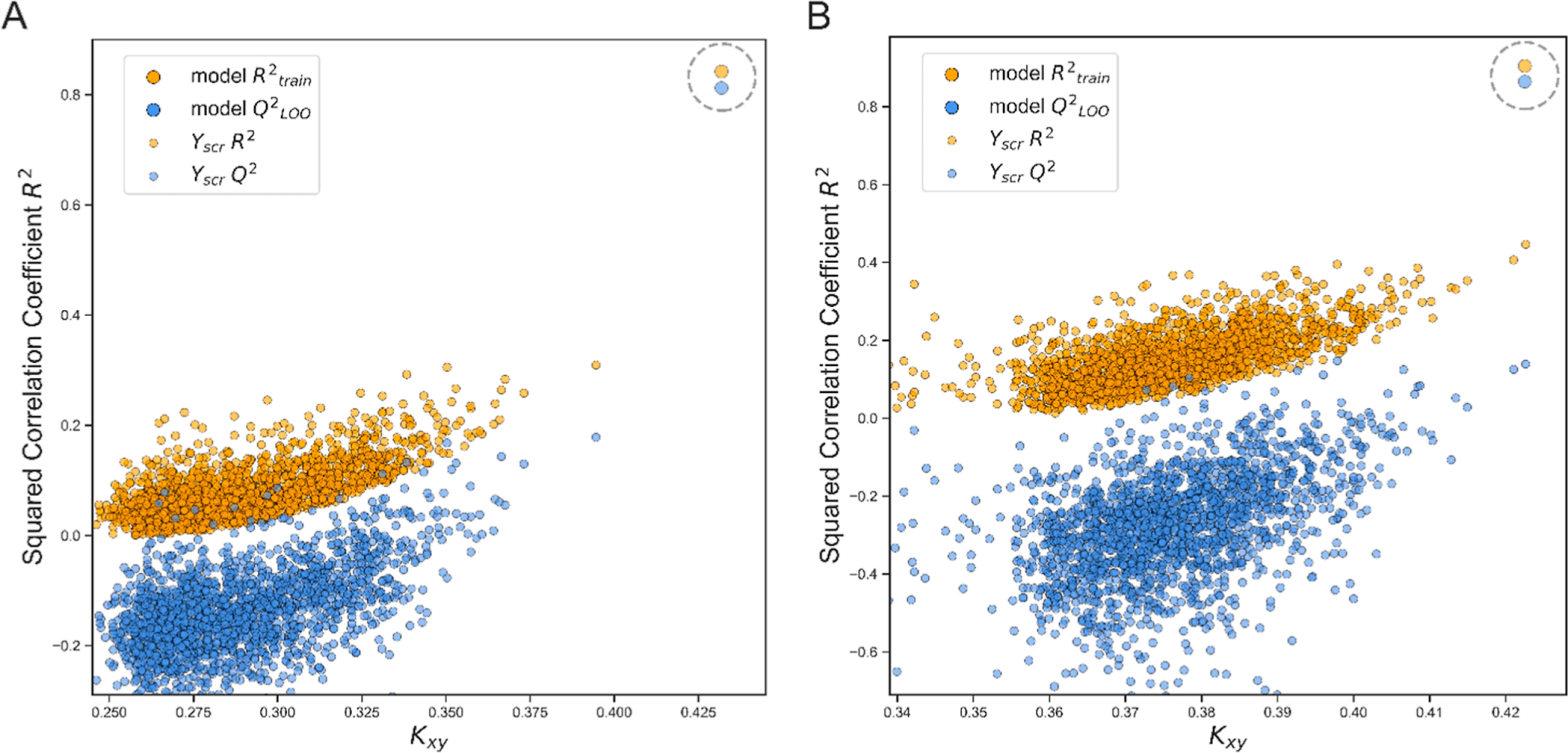
Y-scrambling plots of selected 4-descriptor eq 1 (A) and 8-descriptor eq 2 (B) models.

As a rule, the value of coefficient F indicates the ability of the model to predict the value of the properties in the training set. The large *F* ratio values in both eqs (64.124 and 52.542 for the first and second, respectively) indicate that both equations do an excellent job with predicting ε values. Each equation has an adjusted value of *R*_adj_^2^ 0.829 and 0.888, which denotes a very good correspondence between correlation and data variation. The cross-validated correlation coefficient (*Q*^2^ for eq 1 is equal to 0.813 and *Q*^2^ for eq 2 is eqiual ro 0.865) demonstrates the robustness of the models. The model was further validated by using a *y*-randomization test. The obtained *R*^2^Yscr against the correlation coefficient between the original and shuffled data is shown in Figure 3. It can be seen from Figure 3 that the original models are not due to random correlations; since values of *R*^2^Yscr are significantly low. It is worth noting that model 1 (eq 1) showed much stronger robustness at the *y*-scrambling test than model 2 (eq 2), while both models are quite strong. The calculated results of the values of ε from eqs 1 and 2 for the training and test sets are shown in Table 1 and Figure 1.

**Figure 4:**
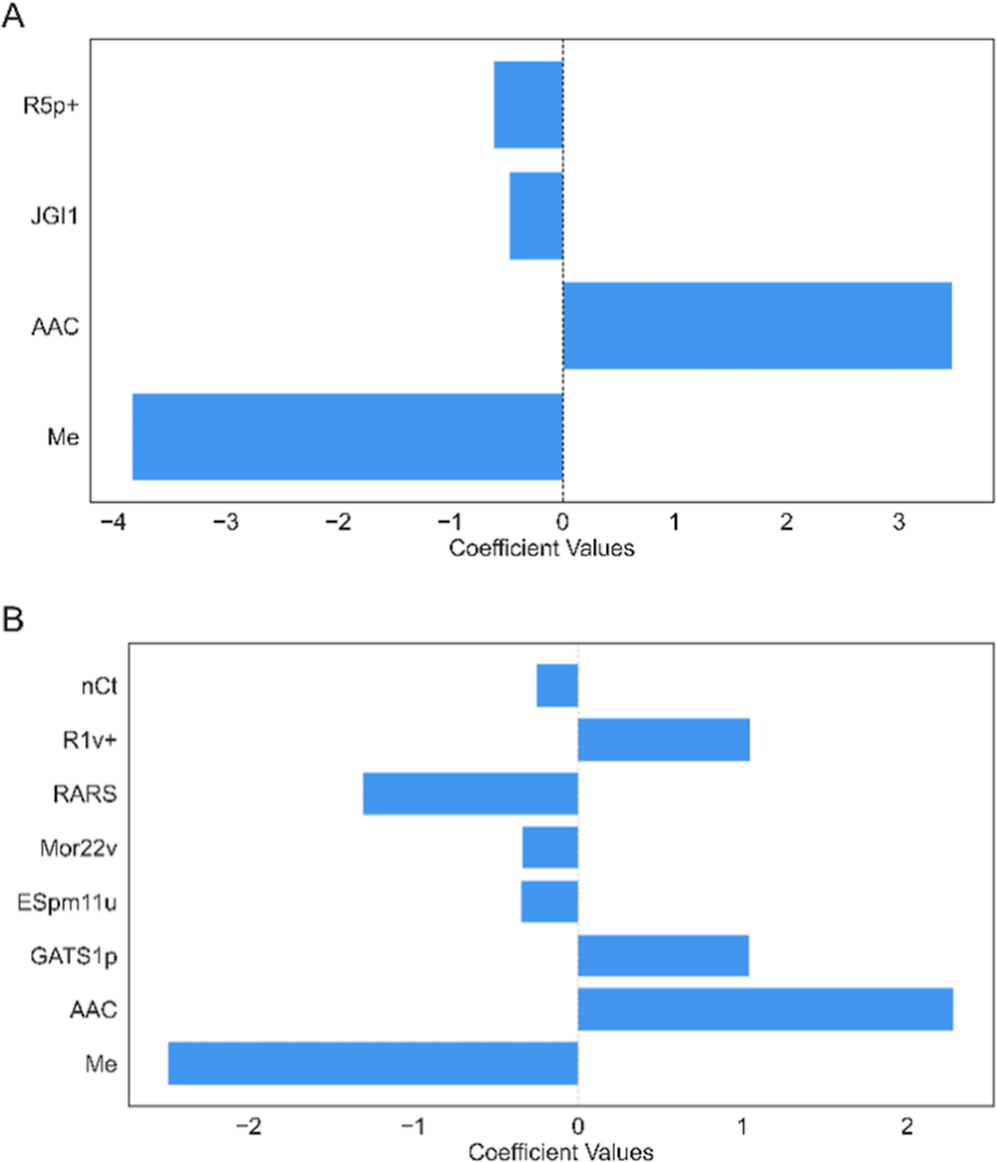
Descriptors contributions to eq 1 (A) and eq 2 (B).

Based on the model selection procedure described earlier, the relative contribution of descriptors to the respective models was determined and shown in Figure 4. The descriptors involved in the model are having the reducing contribution to the model in the following order: for eq 1: Me > AAC > R5p+ > JGI1 and for eq 2: Me > AAC > RARS > R1v+ > GATS1p > ESpm11u > Mor22v > *nCt*.

One of the most important descriptors involved in both equations is the AAC information index. This descriptor contains information about each atom in a molecule by its own atom type, its bond type, and the atom types of its first neighbors. AAC is a measure of atomic composition associated with molecular complexity. When a molecule is larger and its elemental composition is more complex, the value of the descriptor increases. The positive value of this descriptor indicates that polymers with a more complex structure and, accordingly, with a larger value for this descriptor would have larger values of ε. Another descriptor, ESpm11u, is based on the use of bond distances as weights in the diagonal entries of the edge matrix.

It is worth noting that the presented QSPR models can be a good simple way to predict the permittivity of homopolymers. These models can be improved further in future studies by improving the dataset size and variety of polymers. We believe that the results of this study will pave the way for future steps in investigating the electrical conductivity mechanism of polymeric materials.

## Conclusions

In this work, a machine learning-based structure–property relationship model for dielectric constants (ε) based on a diverse set of polymers is developed. A transparent model was obtained with application of the GA-MLRA approach, to get a mechanistically explainable model. This work represents two QSPR models developed based on descriptors computed from monomeric polymer structures. The reliability of the models was validated using several verification methods. The best overall performance is achieved by a four- and eight-descriptor QSAR models, with *R*^2^ values of 0.842/0.715 and 0.905/0.812 for training/test sets, respectively, per each model. The models are suitable for further development of polymers with desired dielectric constants based on chemical structure information of monomers.

## References

[ref1] Huang X.; Jiang P. Core-Shell Structured High-k Polymer Nanocomposites for Energy Storage and Dielectric Applications. Adv. Mater. 2015, 27 (3), 546–554. 10.1002/adma.201401310. 25186029

[ref2] Rao Y.; Wong C. P. Material characterization of a high-dielectric-constant polymer–ceramic composite for embedded capacitor for RF applications. Appl. Polym. Sci. 2004, 92 (4), 2228–2231. 10.1002/app.13690.

[ref3] Dang Z. M.; Yuan J. K.; Yao S. H.; Liao R.; Zhou T.; Li S. T.; Huang W.; Xie C.; Chen P.; Lin Y. Flexible Nanodielectric Materials with High Permittivity for Power Energy Storage. Adv. Mater. 2013, 25 (44), 6334–6365. 10.1002/adma.201301752. 24038139

[ref4] Bicerano J. Prediction of Polymer Properties; Marcel Dekker: New York, 1996.

[ref5] Ku C. C.; Liepins R. Electrical Properties of Polymers; Chemical Principles; Hanser: Munich, 1987.

[ref6] Hougham G.; Tesoro G.; Viehbeck A. Influence of Free Volume Change on the Relative Permittivity and Refractive Index in Fluoropolyimides. Macromolecules 1996, 29, 3453–3456. 10.1021/ma9503423.

[ref7] Schweitzer R. C.; Morris J. B. Improved Quantitative Structure Property Relationships for the Prediction of Dielectric Constants for a Set of Diverse Compounds by Subsetting of the Data Set. J. Chem. Inf. Comput. Sci. 2000, 40, 1253–1261. 10.1021/ci0000070. 11045821

[ref8] Devillers J.; Balaban A. T. Topological Indices and Related Descriptors in QSAR and QSPR; Gordon & Breach: The Netherlands, 1999.

[ref9] Karelson M. Molecular Descriptors in QSAR/QSPR; Wiley Online Library: New York, 2000.

[ref10] Yao X. J.; Wang Y. W.; Zhang X. Y.; Zhang R. S.; Liu M. C.; Hu Z. D.; Fan B. T. Radial basis function neural network-based QSPR for the prediction of critical temperature. Chemom. Intell. Lab. Syst. 2002, 62, 217–225. 10.1016/S0169-7439(02)00017-5.

[ref11] Xu J.; Guo B.; Chen B.; Zhang Q. A QSPR treatment for the thermal stabilities of second-order NLO chromophore molecules. J. Mol. Model. 2005, 12, 65–75. 10.1007/s00894-005-0006-x. 16240094

[ref12] Todeschini R.; Consonni V. Handbook of Molecular Descriptors; Wiley VCH: Weinheim, 2000.

[ref13] Katritzky A. R.; Sild S.; Karelson M. Correlation and Prediction of the Refractive Indices of Polymers by QSPR. J. Chem. Inf. Comput. Sci. 1998, 38, 1171–1176. 10.1021/ci980087w.

[ref14] García-Domenech R.; de Julián-Ortiz J. V. Prediction of Indices of Refraction and Glass Transition Temperatures of Linear Polymers by Using Graph Theoretical Indices. J. Phys. Chem. B 2002, 106, 1501–1507. 10.1021/jp012360u.

[ref15] Xu J.; Chen B.; Zhang Q.; Guo B. Prediction of refractive indices of linear polymers by a four-descriptor QSPR model. Polymer 2004, 45, 8651–8659. 10.1016/j.polymer.2004.10.057.

[ref16] Yu X.; Yi B.; Wang X.; Zhang R. S.; Liu M. C.; Hu Z. D.; Fan B. T. Prediction of refractive index of vinyl polymers by using density functional theory. J. Comput. Chem. 2007, 28, 2336–2341. 10.1002/jcc.20752. 17476666

[ref17] Gao J.; Xu J.; Chen B.; Zhang Q. A quantitative structure-property relationship study for refractive indices of conjugated polymers. J. Mol. Model. 2007, 13, 573–578. 10.1007/s00894-007-0180-0. 17340114

[ref18] Xu J.; Liang H.; Chen B.; Xu W.; Shen X.; Liu H. Linear and nonlinear QSPR models to predict refractive indices of polymers from cyclic dimer structures. Chemom. Intell. Lab. Syst. 2008, 92, 152–156. 10.1016/j.chemolab.2008.02.006.

[ref19] Jabeen F.; Chen M.; Rasulev B.; Ossowski M.; Boudjouk P. Refractive Indices of Diverse Data Set of Polymers: A Computational QSPR Based Study. Comput. Mater. Sci. 2017, 137, 215–224. 10.1016/j.commatsci.2017.05.022.

[ref20] Erickson M. E.; Ngongang M.; Rasulev B. A Refractive Index Study of a Diverse Set of Polymeric Materials by QSPR with Quantum-Chemical and Additive Descriptors. Molecules 2020, 25 (17), 3772 10.3390/molecules25173772. 32825028 PMC7503810

[ref21] Khan M.; Rasulev B.; Roy K. QSPR Modeling of the Refractive Index for Diverse Polymers Using 2D Descriptors. ACS Omega 2018, 3 (10), 13374–13386. 10.1021/acsomega.8b01834. 31458051 PMC6645227

[ref22] Katritzky A. R.; Sild S.; Lobanov V. S.; Karelson M. Quantitative Structure–Property Relationship (QSPR) Correlation of Glass Transition Temperatures of High Molecular Weight Polymers. J. Chem. Inf. Comput. Sci. 1998, 38, 300–304. 10.1021/ci9700687.

[ref23] Mattioni B. E.; Jurs P. C. Prediction of Glass Transition Temperatures from Monomer and Repeat Unit Structure Using Computational Neural Networks. J. Chem. Inf. Comput. Sci. 2002, 42, 232–240. 10.1021/ci010062o. 11911692

[ref24] Cao C.; Lin Y. Correlation between the Glass Transition Temperatures and Repeating Unit Structure for High Molecular Weight Polymers. J. Chem. Inf. Comput. Sci. 2003, 43, 643–650. 10.1021/ci0202990. 12653533

[ref25] Afantitis A.; Melagraki G.; Makridima K.; Alexandridis A.; Sarimveis H.; Iglessi-Markopoulou O. Prediction of high weight polymers glass transition temperature using RBF neural networks. J. Mol. Struct. 2005, 716, 193–198. 10.1016/j.theochem.2004.11.021.

[ref26] Yu X.; Wang X.; Wang H.; Liu A.; Zhang C. Prediction of the glass transition temperatures of styrenic copolymers using a QSPR based on the DFT method. J. Mol. Struct. 2006, 766, 113–117. 10.1016/j.theochem.2006.04.018.

[ref27] Yu X.; Yi B.; Wang X.; Xie Z. Correlation between the glass transition temperatures and multipole moments for polymers. Chem. Phys. 2007, 332, 115–118. 10.1016/j.chemphys.2006.11.029.

[ref28] Bertinetto C.; Duce C.; Micheli A.; Solaro R.; Starita A.; Tiné M. R. Prediction of the glass transition temperature of (meth)acrylic polymers containing phenyl groups by recursive neural network. Polymer 2007, 48, 7121–7129. 10.1016/j.polymer.2007.09.043.

[ref29] Duce C.; Micheli A.; Starita A.; Tiné M. R.; Solaro R. Prediction of the Glass Transition Temperature of Polymer Blends: A Quantitative Structure-property Relationship Approach. Macromol. Rapid Commun. 2006, 27, 711–715. 10.1002/marc.200600026.

[ref30] Yu X. Support vector machine-based QSPR for the prediction of glass transition temperatures of polymers. Fibers Polym. 2010, 11, 757–766. 10.1007/s12221-010-0757-6.

[ref31] Chen M.; Jabeen F.; Rasulev B.; Ossowski M.; Boudjouk P. A Computational Structure-property Relationship Study of Glass Transition Temperatures for a Diverse Set of Polymers. J. Polym. Sci., Part B: Polym. Phys. 2018, 56, 877–885. 10.1002/polb.24602.

[ref32] Petrosyan L. S.; Sizochenko N.; Leszczynski J.; Rasulev B. Modeling of Glass Transition Temperatures for Polymeric Coating Materials: Application of QSPR Mixture-based Approach. Mol. Inf. 2019, 38 (8–9), 1800150 10.1002/minf.201800150. 30945811

[ref33] Karuth A.; Alesadi A.; Xia W.; Rasulev B. Predicting glass transition of amorphous polymers by application of cheminformatics and molecular dynamics simulations. Polymer 2021, 218, 123495 10.1016/j.polymer.2021.123495.

[ref34] Xu J.; Chen B.; Liang H.; Xu W.; Cui W. QSPR Models for the Prediction of Cohesive Energy of Alkanes. Polimery 2009, 54, 19.

[ref35] Ajloo D.; Sharifian A.; Behniafar H. Estimation of Thermal Decomposition Activation Energy for Important Organic Compounds Using QSPR Modeling. Bull. Korean Chem. Soc. 2008, 31, 2009.

[ref36] Yu X.; Wang X.; Wang H.; Li X.; Gao J. Prediction of Solubility Parameters for Polymers by a QSPR Model. QSAR Comb. Sci. 2006, 25, 156–161. 10.1002/qsar.200530138.

[ref37] Rasulev B.; Jabeen F. J.; Stafslien S.; Chisholm B. J.; Bahr J.; Ossowski M.; Boudjouk P. Polymer Coating Materials and Their Fouling Release Activity: A Cheminformatics Approach to Predict Properties. ACS Appl. Mater. Interfaces 2017, 9 (2), 1781–1792. 10.1021/acsami.6b12766. 27982587

[ref38] Cocchi M.; De Benedetti P. G.; Seeber R.; Tassi L.; Ulrici A. Modeling and Prediction by Principal Properties Indexes: 1. Calculation of Dielectric Constant. J. Chem. Inf. Comput. Sci. 1999, 39, 1190–1203. 10.1021/ci9903298.

[ref39] Schweitzer R. C.; Morris J. B. The development of a quantitative structure property relationship (QSPR) for the prediction of dielectric constants using neural networks. Anal. Chim. Acta 1999, 384, 285–303. 10.1016/s0003-2670(98)00781-8.

[ref40] Sild S.; Karelson M. A General QSPR Treatment for Dielectric Constants of Organic Compounds. J. Chem. Inf. Comput. Sci. 2002, 42, 360–367. 10.1021/ci010335f. 11911705

[ref41] Liu J. P.; Wilding W. V.; Giles N. F.; Rowley R. L. A Quantitative Structure Property Relation Correlation of the Dielectric Constant for Organic Chemicals. J. Chem. Eng. Data 2010, 55, 41–45. 10.1021/je900518k.

[ref42] Liu A.; Wang X.; Wang L.; Wang H.; Wang H. Prediction of dielectric constants and glass transition temperatures of polymers by quantitative structure property relationships. Eur. Polym. J. 2007, 43, 989–995. 10.1016/j.eurpolymj.2006.12.029.

[ref43] Golbraikh A.; Tropsha A. Beware of q2!. J. Mol. Graph. Model. 2002, 20, 269–276. 10.1016/S1093-3263(01)00123-1. 11858635

[ref44] Sun L.; Zhou L.; Yu Y.; Lan Y.; Li Z. QSPR Study of Polychlorinated Diphenyl Ethers by Molecular Electronegativity Distance Vector (MEDV-4). Chemosphere 2007, 66, 1039–1051. 10.1016/j.chemosphere.2006.07.013. 16919308

[ref45] Marrero-Ponce Y. Total and Local (Atom and Atom Type) Molecular Quadratic Indices: Significance Interpretation, Comparison to Other Molecular Descriptors, and QSPR/QSAR Applications. Bioorg. Med. Chem. 2004, 12, 6351–6369. 10.1016/j.bmc.2004.09.034. 15556754

[ref46] ACD. Advanced Chemistry Development; ChemSketch, 2014.

[ref47] Turabekova M. A.; Rasulev B. F.; Dzhakhangirov F. N.; Leszczynska D.; Leszczynski J. Aconitum and Delphinium Alkaloids of Curare-Like Activity. QSAR Analysis and Molecular Docking of Alkaloids into AChBP. Eur. J. Med. Chem. 2010, 45 (9), 3885–3894. 10.1016/j.ejmech.2010.05.042. 20594622

[ref48] Juretic D.; Kusic H.; Dionysiou D. D.; Rasulev B.; Loncaric Bozic A. Modeling of Photooxidative Degradation of Aromatics in Water Matrix; Combination of Mechanistic and Structural-Relationship Approach. Chem. Eng. J. 2014, 257, 229–241. 10.1016/j.cej.2014.07.053.

[ref49] Han Y.; Meng Q.; Rasulev B.; May P. S.; Berry M. T.; Kilin D. S. Photofragmentation of the Gas-Phase Lanthanum Isopropylcyclopentadienyl Complex: Computational Modeling vs Experiment. J. Phys. Chem. A 2015, 119 (44), 10838–10848. 10.1021/acs.jpca.5b07209. 26438124

[ref50] Rasulev B. F.; Kušić H.; Leszczynska D.; Leszczynski J.; Koprivanac N. QSAR modeling of acute toxicity on mammals caused by aromatic compounds: the case study using oral LD50 for rats. J. Environ. Monit. 2010, 12 (5), 1037–1044. 10.1039/b919489d. 21491673

[ref51] Hypercube. HyperChem for Windows. Release Version 8.0.7, 2014.

[ref52] Todeschini R.; Consonni V.; Mauri A.; Pavan M. DRAGON-Software for the Calculation of Molecular Descriptors. Web Version 3; DRAGON, 2004.

[ref54] Gramatica P. Principles of QSAR Modeling: Comments and Suggestions from Personal Experience. Int. J. Quant. Struct. Relationships 2020, 5, 61–97.

[ref55] Gramatica P.; Cassani S.; Chirico N. QSARINS-Chem: Insubria datasets and new QSAR/QSPR models for environmental pollutants in QSARINS. J. Comput. Chem. 2014, 35, 1036–1044. 24599647 10.1002/jcc.23576

[ref53] Gharagheizi F.; Eslamimanesh A.; Ilani-Kashkouli P.; Mohammadi A. H.; Richon D. QSPR molecular approach for representation/prediction of very large vapor pressure dataset. Chem. Eng. Sci. 2012, 76, 99–107. 10.1016/j.ces.2012.03.033.

